# Anti-glomerular basement membrane disease in children: can Sars-Cov-2 be a trigger?

**DOI:** 10.1590/2175-8239-JBN-2023-0120en

**Published:** 2024-03-15

**Authors:** André Costa Azevedo, Ricardo Domingos Grilo, Ana Patrícia Rodrigues, Ana Losa, Liane Correia-Costa, Ana Teixeira, Liliana Rocha, Paula Matos, Teresa Costa, Maria Sameiro Faria, Conceição Mota

**Affiliations:** 1 Centro Hospitalar Universitário de Santo António, Centro Materno-Infantil do Norte Albino Aroso, Serviço de Pediatria Médica, Unidade de Nefrologia, Porto, Portugal. Porto, Portugal.; 2 Unidade Local de Saúde do Alto Minho, Serviço de Pediatria, Viana do Castelo, Portugal.; 3 Hospital do Espírito Santo de Évora, Departamento da Mulher e da Criança, Serviço de Pediatria, Évora, Portugal.; 4 Centro Hospitalar Universitário de Santo António, Serviço de Anatomia Patológica, Porto, Portugal.; 5 Centro Hospitalar Universitário de Santo António, Centro Materno-Infantil do Norte Albino Aroso, Serviço de Pediatria Médica, Porto, Portugal.; 6 Universidade do Porto, Instituto de Ciências Biomédicas Abel Salazar, Porto, Portugal.; 7 Universidade do Porto, Instituto de Saúde Pública, Unidade de Investigação em Epidemiologia, Porto, Portugal.; 8 Universidade do Porto, ITR-Laboratório para a Investigação Integrativa e Translacional em Saúde Populacional, Porto, Portugal.; 9 Universidade NOVA de Lisboa, Unidade de Ciências Biomoleculares Aplicadas, Lisboa, Portugal.

## Case Presentation

**Figure 1:**
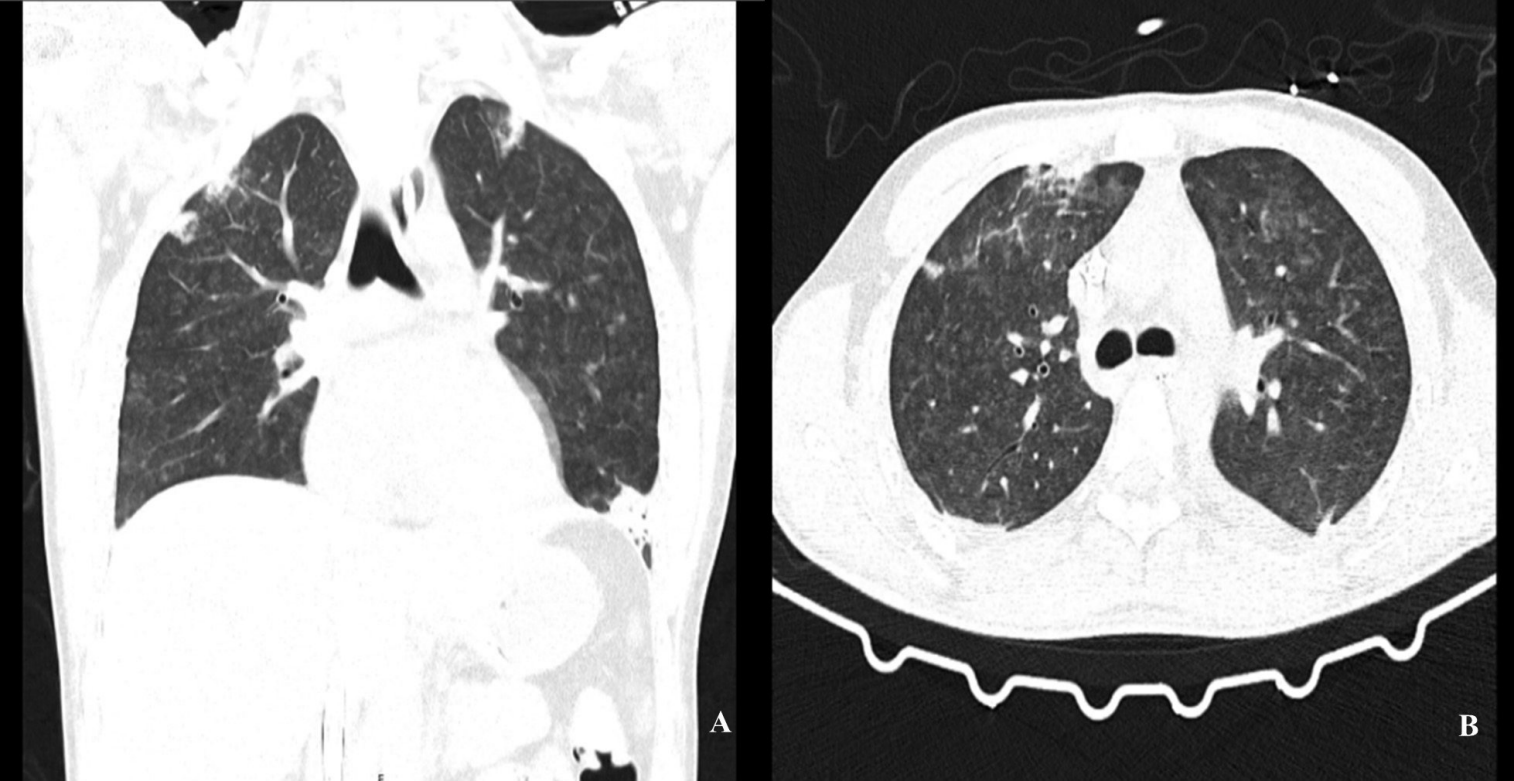
Angio-CT scan showing consolidation with atelectasis centered in the apical segments of the lungs.

A 17-year-old Caucasian male, smoker (20 cigarettes/day), without no other drug or alcohol consumption, previously vaccinated with two doses of SARS-CoV-2 vaccine and with an unremarkable medical history, presented to the pediatric emergency department with an 11-day history of dyspnea on mild exertion, thoracalgia, and bloody sputum for the previous 4 days. At presentation, examination revealed cutaneous pallor, respiratory distress, hypoxemia (saturation of 87% on room air), and crackles on pulmonary auscultation. Initial laboratory tests (Table 1) were positive for leukocytosis (14340/μL), neutrophilia (10700/μL), elevated C-reactive protein (148.4 mg/L), and normal kidney function (serum creatinine of 0.74 mg/dL/dL and serum urea of 24 mg/dL). Due to significant respiratory distress, computed tomography angiography (Angio-CT) of the thorax was performed, revealing multiple micronodular opacities, and pulmonary embolism was excluded. Polymerase chain reaction (PCR) testing for SARS-CoV-2 was also performed, and a positive result was obtained. The patient was admitted on supplemental oxygen, but respiratory distress worsened to the point of requiring ventilatory support. He was transferred to the pediatric intensive care unit (PICU) for further care and management. During his stay in the PICU, angio-CT was repeated, demonstrating a consolidation with atelectasis centered in the apical and posterior segments of the lungs (shown in Figure 1). The remaining parenchyma was airy and filled with multiple diffuse, centrilobular, micronodular opacities that only spared the pleural surface and fissures, a pattern that suggested subacute hypersensitivity pneumonitis. He was started on intravenous antibiotics (piperacillin-tazobactam was switched to imipenem and vancomycin due to clinical worsening and an increase in inflammatory markers). Immunological studies were performed and proved negative for the following antibodies: anti-nuclear (ANA), anti-neutrophil cytoplasmatic (ANCA), anti-cardiolipin, anti-extractable nuclear antigen (ENA), and rheumatoid factor. Blood cultures and bacteriological and mycological cultures of respiratory secretions were negative. Interferon Gamma Release Assay (IGRA) was also negative. The patient’s clinical condition deteriorated, and showed to be refractory to mechanical ventilation. He was placed on extracorporeal membrane oxygenation (ECMO) and started on high-dose steroid therapy. Bronchoscopy and bronchoalveolar lavage showed neutrophilia and mild alveolar hemorrhage, and culture exams were negative. Clinically, the patient’s condition improved, steroid doses were progressively decreased, and ECMO was stopped. After ECMO decannulation, cervical doppler was performed, revealing a thrombus filling 20% of the lumen of the right internal jugular vein. Doppler findings of the lower limb veins were normal. Anticoagulation was initiated with apixaban. During this hospitalization, serial blood samples showed slow normalization of the inflammatory markers’ levels. There were no further episodes of hemoptysis and kidney function remained normal, with a maximum serum creatinine level of 0.63 mg/dL, and 0.51 mg/dL at discharge to a rehabilitation unit (Table 1) due to severe disuse myopathy. During his stay in the rehabilitation unit, the patient remained hemodynamically stable with normal urinary output until 2 months after admission, when he developed fever and gross hematuria. The patient was immediately transferred to the Pediatric Nephrology Unit.

**Table 1:** Evolution of the most important laboratory exams. anca – anti-neutrophil cytoplasm antibody; ana – antinuclear antibodies; gbm – glomerular basement membrane; mpo – myeloperoxidase; pr3 – proteinase 3

		Pediatric Intensive Care Unit		Pediatric Nephrology Unit			
		*Admission*	*Discharge*	*Admission*	*D4*	*D14(1 week after first plasmapheresis)*	*Discharge*
**Hemoglobin** (13 – 17 g/dL)		13.1	9.1	10.9	10.8	8.1	9.2
**White blood count (WBC)** (4.5 – 11/µL)		14.34	6.74	9.71	16.65	18.5	8.30
**Platelet count** (150 – 400 × 1000/µL)		320	188	299	459	204	288
**Urea** (10 – 50 mg/dL)		24	20	52	148	88	169
**Creatinine** (0.7 – 1.2 mg/dL)		0.74	0.51	2.97	8.31	5.93	5.36
**Albumin** (3.5 – 5.0 g/dL)		4.12	3.16	3.2	3.13	3.69	4.49
**Sodium** (135 – 145 mmol/L)		136	142	139	140	139	145
**Potassium** (3.5 – 5.0 mmol/L)		3.7	3.7	4.27	6.06	4.68	5.07
**Phosphorus** (0.87 – 1.45 mmol/L)		1.33	3.7	1.48	1.31	1.50	0.81
**Calcium** (2.10 – 2.55 mmol/L)		2.18	2.24	2.28	2.30	2.12	2.22
**Anti-GBM** (< 10 U/mL)		–	–	–	1139	659	14
**ANCA** (< 1/20)		Negative	–	–	1/320 (atypical P-ANCA)	1/20 (atypical P-ANCA)	–
**Anti-PR3** (< 20 U)		< 2.3	–	–	< 2.3	< 2.3	–
**Anti-MPO** (< 20 U)		< 2.3	–	–	< 2.3	< 2.3	–
**ANA**		Negative	–	–	Negative	–	–
**Urine Analysis**	**pH** (4.8 – 7.4)	8.0	–	5.5	5.0	–	–
	**Density** (1.015 – 1.025)	1.016	–	1.015	1.009	–	–
	**Leukocytes** (0 – 2 /field 400x)	0 – 2	–	0 – 2	2 – 5	–	–
	**Erythrocytes** (0 – 2 /field 400x)	0 – 2	–	> 50	> 50	–	–
	**Casts**	None	–	None	None	–	–
**Proteins in a single urine sample** (< 0.15 g/L)		< 0.15	< 0.15	0.95	0.48	–	–

## Pediatric Nephrology Unit Course

Upon admission, the patient revealed cutaneous pallor and crackles in the right hemithorax. Initial work-up (Table 1) was relevant for elevated serum creatinine (2.97 mg/dL), serum urea (52 mg/dL), and elevated C-reactive protein (282.9 mg/L). Chest radiography revealed no consolidation or pleural effusion. Renal ultrasound showed slightly enlarged kidneys (right and left kidney around 13.5 cm) with increased cortical echogenicity and reduced corticomedullary differentiation. An immunological study was performed again including ANA, ANCA, and anti-GBM antibodies. Blood and urine cultures were obtained and proved to be negative. The anticoagulants were then suspended. Upon admission, he was started on intravenous antibiotics and oral therapy with high doses of steroids (prednisone 60 mg/day). Serial blood samples showed deterioration of renal function: serum creatinine and urea levels reached a maximum of 8.31 mg/dL and 148 mg/dL, respectively. The serum potassium level increased to a maximum of 6.06 mmol/L. The patient was oliguric at admission and progressively became anuric from the first day onwards. Emergency hemodialysis was initiated to correct electrolyte imbalances.

## Histopathology

**Figure 2:**
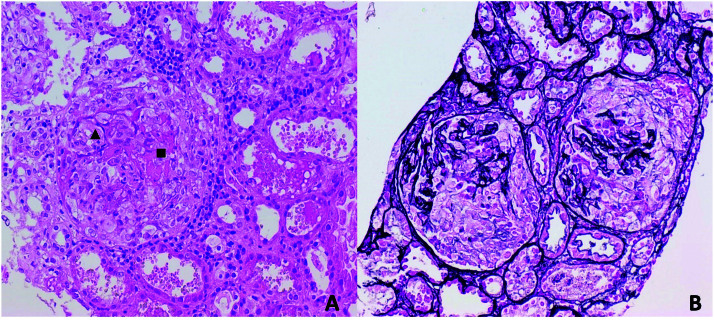
Large cellular crescents occupy all glomeruli in this biopsy. Focal fibrinoid necrosis (■) and disruption of Bowman capsule (▲) are associated with interstitial inflammation (2A, HE, 200x). The glomerular basement membrane is commonly fragmented in glomeruli with crescents. This can be seen in the silver-stained section (2B, silver-stain, 200x).

**Figure 3:**
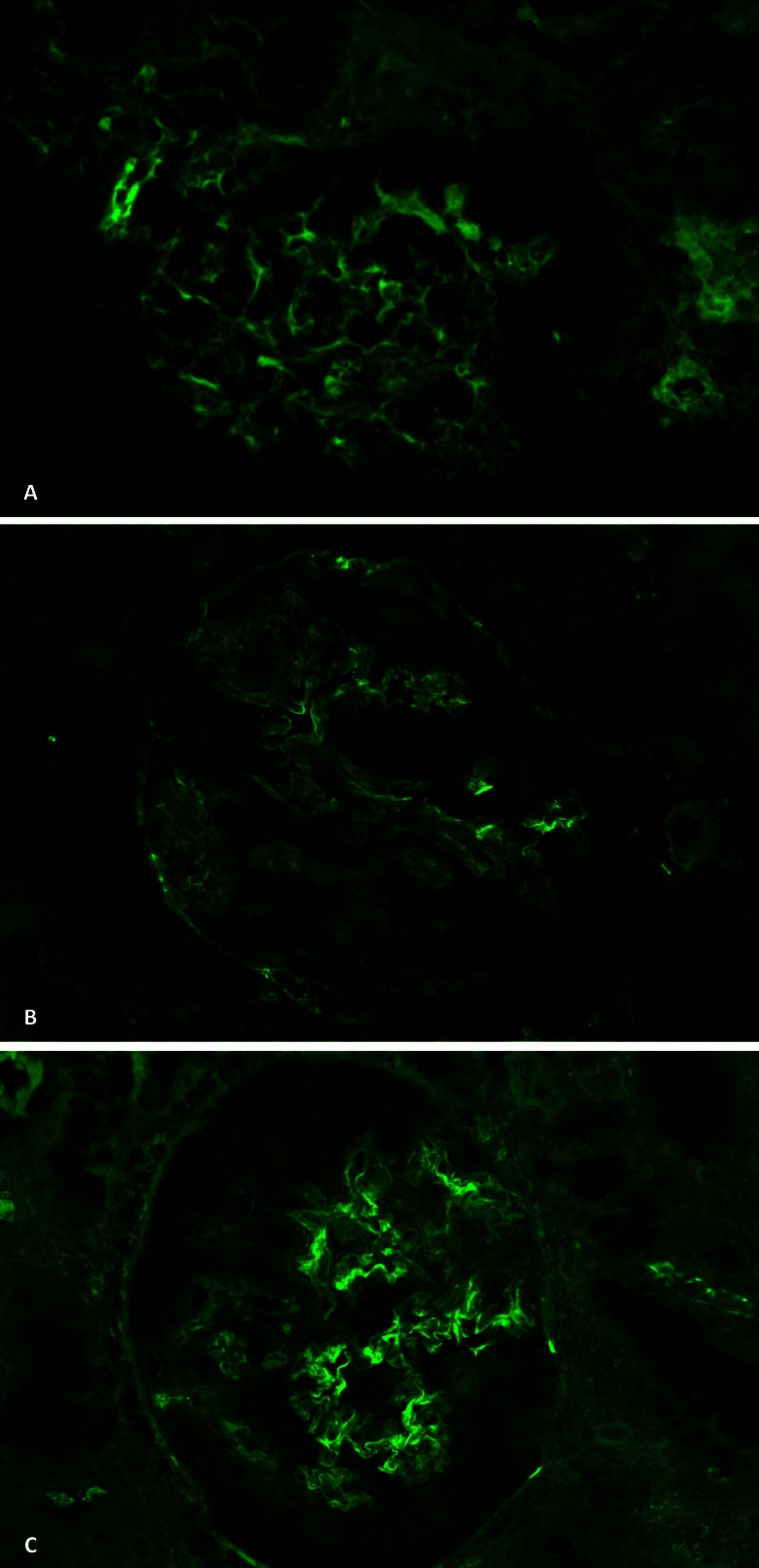
Immunofluorescence showing IgG (A), IgM (B), and C3 (C) deposition over the glomerular basement membrane.

On day 4 after admission, renal biopsy was performed. All 18 visualized glomeruli showed fibrocellular crescents and fibrinoid necrosis foci (shown in Figure 2). Crescents were associated to capillary walls’ rupture. The interstitium was involved by inflammatory infiltrate composed of lymphomononuclear cells and scattered eosinophils. Immunofluorescence showed linear glomerular capillary staining for C3, IgG, and IgM, all 2+ on a scale of 0 to 3+ (shown in Figure 3). Renal biopsy findings were consistent with GBM-mediated crescentic glomerulonephritis. This corresponds to a crescentic class in the histopathological classification of Berden.

## Final Diagnosis

Renal biopsy findings were in line with the high titers of anti-GBM-antibody (1139U/mL) and atypical P-ANCA (1/320). Bronchoscopy was performed, and alveolar hemorrhage was confirmed. The patient was diagnosed with anti-glomerular basement membrane disease (anti-GBM).

## Treatment

Once the biopsy results and high anti-GBM titers confirmed the diagnosis of anti-GBM disease, the patient was started on a high-dose bolus of methylprednisolone for 3 days, followed by oral high-dose steroid therapy and daily sessions of plasmapheresis (1 – 1.5 total plasma volume exchanged with 5% albumin replacement). Furthermore, oral cyclophosphamide was initiated (2 mg/kg/dose adjusted for renal function). On day 20 after admission, the patient’s clinical condition worsened, new onset of hemoptysis associated with hypoxemia was observed, and he was started on supplemental oxygen (maximum 1 L/minute). In order to prevent progression of the alveolar disease and decrease the persistently high titers of anti-GBM antibodies, 4 infusions of rituximab (375 mg/m^2^; 4 consecutive weeks) associated with intravenous methylprednisolone bolus were used as an alternate immunosuppressive agent, and cyclophosphamide was suspended after 18 days of treatment. Anti-GBM antibody titers started to decrease, and plasmapheresis sessions were switched to alternate days after 22 daily sessions. Anti-GBM levels reached almost normal values (14 U/mL), and pulmonary symptoms resolved; however, as expected due to the severity of the presentation, kidney function did not recover, and regular outpatient hemodialysis was maintained. The patient was expected to be placed on the kidney transplant waiting list after the stabilization of the underlying disease.

## Discussion

Anti-GBM is an extremely rare cause of glomerulonephritis, accounting for 0.4% of all pediatric chronic kidney disease stage 5 in children. It has a bimodal distribution with the first peak affecting mainly males in their teens and twenties and a second peak affecting older people (more than 60 years old)^1^. The age range mentioned is in line with the patient age in this case report.

Most patients present with rapidly progressive glomerulonephritis, and concomitant alveolar hemorrhage may occur in some cases^1,2^. Kidney manifestations are characterized by acute kidney injury with urinalysis showing proteinuria, usually not in the nephrotic range, and sediment with dysmorphic red cells, white cells, and red cells and granular casts^3^. On the other hand, pulmonary involvement is associated to shortness of breath, cough, hemoptysis, and pulmonary infiltrates on chest radiograph^4^. A small proportion of patients have isolated pulmonary findings, which are more likely in younger patients^1^. In this case, the patient’s first symptom was hemoptysis, which could already be a sign of the disease.

The etiology is often unknown, but upper and lower tract infections may trigger the disease. Clustering of cases has been observed in influenza A outbreaks in the past^2,5^. Early diagnosis is important in terms of renal function recovering. The diagnosis of anti-GBM relies on serological testing for anti-GBM antibodies^3,4^. Percutaneous kidney biopsy establishes the diagnosis and helps determine the probability of recovery based on the percentage of crescents identified^3,4^.

Co-presentation with both ANCA and anti-GBM antibodies (double-positives) is rare. In fact, since it was first described in 1980, the boundary between ANCA-associated vasculitis and anti-GBM disease has been blurred^6^. Whether the diagnosis is ANCA-associated vasculitis with anti-GBM antibodies or anti-GBM disease with ANCA relies on the demonstration of linear IgG deposition on the GBM^6^. Furthermore, a positive PR3-ANCA or MPO-ANCA result is highly suspicious for the diagnosis of ANCA-associated vasculitis; however, other atypical ANCA patterns may also be detected in a wide range of inflammatory and auto-immune diseases^7^. In this particular case, immunofluorescence shows the IgG linear deposit on GBM, and enzyme-linked immunosorbent assay (ELISA) was negative for MPO and PR3, confirming the diagnosis.

McAdoo et al.^8^ analyzed the clinical features and long-term outcomes of a large cohort of 568 patients with ANCA-associated vasculitis, 41 patients with anti-GBM disease, and 37 double-positive patients with ANCA and anti-GBM disease from four European centers. Double positive patients demonstrated to have a hybrid disease phenotype, sharing ANCA-associated vasculitis characteristics (older age distribution and longer symptom duration before diagnosis), and anti-GBM disease (lung hemorrhage at presentation and severe renal disease)^8^. Although in this case, anti-GBM were not initially looked for and ANCA was negative may have started before the overt clinical presentation and may have been hidden by the coronavirus disease 2019 (COVID-19) infection and treatment.

Anti-GBM disease treatment includes plasmapheresis in order to remove circulating antibodies. Plasmapheresis has been demonstrated to be beneficial for renal function prognosis when the initial serum creatinine is below 5.7 mg/dL^9^. Immunosuppressive therapy comprises high doses of steroids and cyclophosphamide, which reduce antibody production. Rituximab has also been reported as an alternative, particularly in cases of refractory disease or in patients presenting with serious adverse events with cyclophosphamide^10^.

Recently, an unexpected number of cases of anti-GBM have been reported during the COVID-19 pandemic, and for that reason, an association between this condition and SARS-CoV-2 has been hypothesized^11–14^. The majority of the cases link COVID-19 and new-onset of the autoimmune disease in adults, making its presence rare in the pediatric patients. A total of eight new cases were diagnosed in adults in North West London, United Kingdom, between December 2019 and April 2020, which corresponds to a fivefold increase in the disease^11^. Moreover, a study in India showed a 68% increase in anti-GBM disease in biopsied patients with acute kidney injury compared with pre-COVID-19 data^12^. Another case series showed temporal clustering in patients with positive IgM antibodies for SARS-CoV-2 and the diagnosis of anti-GBM disease^13^. Furthermore, SARS-CoV-2 infection has also been implicated in disease recurrence, as it has been reported in a 31-year-old woman^14^.

Although the relationship between anti-GBM and SARS-CoV-2 remains unknown, the potential of viral infections to trigger autoimmunity, including progressive forms of glomerulonephritis, has been proven. In the present case, the patient had hemoptysis and tested positive for SARS-CoV-2 at presentation, although no renal involvement was evident at that time. Hemoptysis is a rare symptom of COVID-19; however, few cases have been described in the literfature^15^. On the other hand, alveolar hemorrhage, although less common, can also be a symptom of anti-GBM^15^. Hence, in this patient, SARS-CoV-2 may have been the trigger or just an innocent bystander of the disease. However, the causal relationship remains speculative and further studies are needed to better define this association.
